# Free radical theory of autoimmunity

**DOI:** 10.1186/1742-4682-3-22

**Published:** 2006-06-07

**Authors:** Subburaj Kannan

**Affiliations:** 1 DNA Repair & Drug Resistance Group, Departments of Microbiology and Immunology, School of Medicine, University of Texas Medical Branch, Galveston, Texas 77555-0609, USA

## Abstract

**Background:**

Despite great advances in clinical oncology, the molecular mechanisms underlying the failure of chemotherapeutic intervention in treating lymphoproliferative and related disorders are not well understood.

**Hypothesis:**

A hypothetical scheme to explain the damage induced by chemotherapy and associated chronic oxidative stress is proposed on the basis of published literature, experimental data and anecdotal observations. Brief accounts of multidrug resistance, lymphoid malignancy, the cellular and molecular basis of autoimmunity and chronic oxidative stress are assembled to form a basis for the hypothesis and to indicate the likelihood that it is valid *in vivo*.

**Conclusion:**

The argument set forward in this article suggests a possible mechanism for the development of autoimmunity. According to this view, the various sorts of damage induced by chemotherapy have a role in the pattern of drug resistance, which is associated with the initiation of autoimmunity.

## Background: review of the literature

### Multi-drug resistance: *a multi-step process*

After exposure to chemotherapeutic drugs, lymphoid cells develop along two distinct pathways. First, a cell population susceptible to the drugs dies by apoptosis or necrosis, depending on the severity of treatment. Secondly, a few cells evolve one or more mechanisms for survival, resisting the damage inflicted by the drugs. It is well known that chemotherapeutic drugs induce tumor cell death via apoptosis through DNA damage, and, in particular, activation of proteolytic enzymes involved in programmed cell death. When one drug fails, various others are tried as parts of a therapeutic regimen. Such drugs kill cancer cells by increasing their sensitivity via alterations in internal mechanisms, a desired outcome for effective chemotherapy. Some tumor cells evolve mechanisms, as yet poorly understood, by which they acquire resistance to structurally and functionally unrelated drugs; this is referred to as multi-drug resistance.

### Multi-drug resistance: *a selective adaptation mechanism*

Distinct factors contributing to the formation of tumorigenic phenotypes ensure that each malignant cell is unique in terms of activation of oncogenes and inactivation of tumor suppressor genes. Drug-exposed tumor cells are subjected to sustained to oxidative stress and become tolerant to it. During this time window, selection pressure imposed by the chemotherapeutic drugs causes the selective overgrowth of cells that can withstand them. It is also possible that normal, but susceptible, cells may acquire drug resistance by cellular overgrowth in their neighborhood [1].

### Multi-drug resistance: *an intrinsic or acquired phenomenon*

Development of drug resistance could be either intrinsic or acquired during neoplasia formation. Intrinsic resistance is possibly an inherent property of the species, developed during the course of evolution. Acquired drug resistance possibly originates in the host because of one or more of the following factors: **1. **reduced absorption of the specific drug; **2. **delayed/expedited rate of metabolism by the specific organ involved; **3. **loss of drug accumulation mechanism (decreased import); **4. **increased drug elimination (increased export) (e.g. multi-drug resistance in cancer cells); **5. **conversion of active drug to an inactive form (e.g. penicillinase, insecticide resistance) or to a prodrug no longer converted to its active form (e.g. resistance to purine analogues in cancer cells); **6. **elimination of target (e.g. induction of alternative pathway) or alteration of target's affinity for the drug; **7. **overproduction of target (e.g. gene amplification); **8. **accumulation of metabolite antagonistic to drug (e.g. PABA overproduction by Pneumococci) [2].

### Multi-drug resistance: *evolution by inhibition of apoptosis*

These factors contribute towards reducing the level of the drug in the serum. Other factors contributing to the evolution of drug resistance and inhibition of apoptosis may include: tolerance to the drug effects; failure and/or lack of delivery of a given drug to the tumor site (owing to size or location of the tumor, or low absorption rate of a high molecular weight drug); and non- specific interactions of drugs with healthy cells [3,4]. As a result, each malignant cell is unique in terms of activation of oncogenes and inactivation of tumor suppressor genes and hence in the tumorigenic phenotypes to which it can give rise; any given tumor cell population becomes heterogeneous [5]. Although many studies have demonstrated the critical role of anti-apoptotic components including Bcl-2, Bcl-xL and Mcl-1, and proapoptotic components such as Bax, Bak and Bad, in the evolution of multi-drug resistance, the underlying molecular mechanism is not clear at present. Overexpression of Bcl-2, Bcl-xL or Mcl-1 has been shown to prevent drug-induced apoptosis in several cell lines [6,7].

### Multi-drug resistance: *role of epigenetic mechanism(s)*

It has been suggested that drug resistance is implicitly mediated via epigenetic changes in the form of altered gene expression induced by transacting factors, and is definitely not due to alteration of the tumor cell genome. However, the DNA double strand breaks (dsbs) are considered responsible for drug toxicity and are linked to cell death, mostly via apoptosis [8,9]. Drug-sensitive cells exposed to alkylating agents manifest a sustained increase in reactive oxygen species (ROS) levels along with DNA dsbs. ROS and dsbs are suggested causes of drug sensitive tumor cell death via apoptosis. Furthermore, after a period of time without exposure to alkylating agents, drug resistant cells in culture become sensitive and die via apoptosis. It is tempting to speculate that the drug resistance observed *in vitro *is a transient or evanescent phenomenon. This may not be the case in the *in vivo *or clinical scenario, which would severely limit the ability to correlate *in vitro *findings with clinical manifestations **(Kannan, unpublished data).**

### Multi-drug resistance: *possible role of DNA damage-dependent mechanisms*

In addition, acquired resistance to the alkylating family of drugs has been attributed to such factors as increased expression of glutathione-S-transferase (GST) and changes associated with signaling events upstream of the site of the action of Bcl-2 family members. It is implied that genes conferring protection from apoptosis are up-regulated in the surviving cells and confer drug resistance. The steps involved in the formation of resistance to alkylating agents involve the following sequence: cellular uptake of the drug [10], conversion to the active form, formation of DNA-mono adducts, cross-link formation, and detoxification of the free intracellular drug or its reactive metabolite, e.g. by conjugation with reduced glutathione by GST [4].

### Multi-drug resistance: *possible role of DNA damage-independent mechanisms*

However, it has also been proposed that drug resistance can evolve independently of DNA damage and repair [11]. Oxidative stress-induced DNA damage could be overcome in drug-sensitive tumor cells, but it probably occurs through the loss of appropriate antiapoptotic genes, so the sensitive cells undergo apoptosis. Drug-resistant cells overcome oxidative stress by efficiently repairing the as-yet-unknown extent of damage in genomic DNA, resulting in a drug resistance genotype. In terms of DNA repair, an alkylating agent-resistant phenotype of B-cell chronic lymphocytic leukemia has been attributed to rapid inter-strand, cross-link repair, and also to upregulation of double-strand repair proteins with increased formation of nuclear foci [12].

Owing to the sustained accumulation of DNA strand breaks and impairment of the DNA double-strand break repair machinery, such as homologous recombination (HR) and non-homologous endjoining (NHEJ), along with genomic instability to the alkylating agents, the resistant cells undergo apoptosis [13]. In a comparative study using variants of human ovarian carcinoma cells sensitive or insensitive to alkylating agents, it was found that increased levels of anti-apoptotic proteins prevent the drug-resistant cells dying from apoptosis. In contrast, by producing increased levels of pro-apoptotic proteins, the drug-sensitive cells were rendered apoptotic. The sensitive cells were shown to undergo more free radical formation and genomic DNA damage with the impairment of DNA damage repair mechanisms [[14-16]; Kannan, unpublished observations].

### Malignant lymphocyte disorders

Lymphocyte malignancies encompass the development of lymphocyte neoplasms from cells at a stage before their differentiation to T- and B- cells; i.e. either primordial or differentiated stem cells. In particular, acute lymphocytic leukemias are known to originate from the primitive lymphoid stem cells that normally differentiate into T- or B-cell phenotypes, whereas chronic lymphocytic leukemias arise from a well- differentiated B-cell progenitor. It is also known that multiple myelomas are likely to originate from B cells at a later stage of maturation. Depending on the various regulatory molecules involved in stem cell development, the ensuing lymphocytic disease might be of several kinds: **I**. hairy-cell leukemia, **II**. prolymphocytic leukemia, **III. **natural killer-cell large granular lymphocytc leukemia and **IV. **plasmacytoma.

Both B-cell and T-cell neoplasms constitute a multiplicity of disorders depending on the developmental stage (see table 96-1 in[17]). Secretions of monoclonal proteins (a distinctive class of immunoglobulins) are an essential feature of both neoplastic transformation and clonal proliferation in B cells. Owing to the increase in secretory protein levels in the circulation, the viscosity of the blood is increased and erythrocytes aggregate, causing a hyper-viscosity syndrome [18]. Moreover, immunoglobulins that precipitate below 37°C are known to cause Raynaud syndrome, skin ulcerations, purpura, digital infarction and gangrene. Deposition of immune complexes in the glomerular tufts is a critical factor in the evolution of a spectrum of renal diseases. Formation and accumulation of excess immunoglobulin heavy chains in plasma cell myelomas causes amyloids, resulting in primary amyloidosis.

The generation of immunoglobulins that recognize self antigens, referred to as auto-reactive antibodies, during B-cell neoplasia is known to cause autoimmune thrombocytopenia and possibly autoimmune neutropenia. Antibodies directed against tissue proteins are factors in the etiopathogenesis of autoimmune thyroiditis, adrenalitis, encephalitis, and, conceivably, peripheral neuropathies. Although neuropathy is not a common sequela, organomegaly, endocrinopathy or POEMS syndrome are potential outcomes (reviewed in [17]). Malignant B-cell infiltration into bone marrow suppresses hemopoiesis, causing anemia, granulocytopenia and possibly thrombocytopenia. While combinations of proliferating and infiltrating malignant B-cells are a significant factor in the genesis of splenomegaly and lymphadenopathy of superficial or deep lymph nodes, malignant B-cells in prolymphocytic or hairy cell leukemia, after infiltrating the bone marrow and spleen, may cause spleen enlargement (reviewed in [17]).

Cutaneous T-cell lymphomas are associated with elevated levels of Th-2-type-associated cytokines such as IL-2, IL-4, IL-5, IL-10, IL13, IFN-γ, TNF-β, TNF-α and GM-CSF, which might compound the occurrence of eosinophilia and eosinophilic pneumonia [19]. The neoplastic plasma cells in multiple myeloma secrete IL-1, causing the stimulation of osteoclast proliferation, oteolysis, severe bone pain and pathological fractures [20]. Moreover, in lymphoma-associated hemo-phagocytic syndrome, excessive secretion of IL-1 might play a role in the inappropriate secretion of antidiuretic hormone [21]. It has been suggested that the uncontrolled extra-renal production of calcitriol is the major humoral mediator of hypercalcemia in both Hodgkin's disease and non-Hodgkin's lymphomas [22]. Lymphocytic malignancies are known to be sensitive to cytotoxic drugs, causing hyperuricosuria, hyperkalemia and hyperphosphatemia. These abnormalities result from metabolic disruption and are collectively referred to as "tumor lysis syndrome" [23].

Cutaneous T-cell lymphomas encompass malignant cells that home to skin, occasionally producing desquamating erythroderma, as observed in Sézary syndrome, nodular infiltrative lesions, and, in specific incidences, mycosis fungoides. Lymphocytic leukemia and lymphoblastic lymphomas of T-cell origin are associated with the enlargement of mediastinal regions. B-cell lymphomas have frequently been observed in bones, bowel, kidneys, lungs, heart, joints, endocrine and salivary glands and less frequently in the extra-nodal regions. Marginal zone B-cell lymphomas of the mucosa-associated lymphoid tissue (MALT) type have been recorded mostly in the stomach and also in the salivary glands, though extra-nodal origin is a possibility, as indicated by columnar or cuboidal epithelium in staging (reviewed in [17]).

### Loss of self-tolerance and onset of autoimmunity: a muti-factorial phenomenon

Among the foremost risk factors for the development of autoimmune diseases are polymorphisms among genes regulating the onset of the self-tolerance and immune regulation – *(autoimmune regulator (AIRE)*, the T cell immunoglobulin and mucin-domain-containing (TIM) gene family, and cytolytic T lymphocyte-associated antigen 4 (CTLA-4)) – which have a significant role in sustaining self-tolerance and in the onset of autoimmunity (reviewed in [24-26]). *AIRE *null mice develop multi-organ failure because of the specific reduction in ectopic transcription of genes encoding peripheral antigens. On the basis of these findings it has been suggested that thymically-imposed "central" (self) tolerance plays a pivotal role in the in genesis of autoimmunity [27].

### Cellular basis of antigen presentation and cross-presentation: role of MHC and T-cells

In particular, the MHC haplotype contributes either by enabling peptide epitopes to be presented in the periphery, increasing T cell activation, or by aborting the presentation of self(host)-antigens in the thymus. As a result, more aggressive T cells or fewer regulatory T (Treg) cells are formed in the host. It is known that Th1-type responses such as interferon-γ [IFN-γ] and interleukin 12 [IL-12] are associated with destructive autoimmune responses, while Th2-type responses (IL-4, IL-5, IL-13) counter-regulate cell-mediated autoimmune processes [28].

Helper T (CD4+) cells recognize peptides presented by MHC class II molecules, whereas cytotoxic T cells (CTLs/CD8) recognize peptides presented by MHC class I. MHC class II molecules present peptides originating from exogenous sources that enter the cell by endocytosis. MHC class I molecules present antigens of endogenous origin, which are synthesized within the cells. Dendritic cells also process exogenous antigens into the MHC class I pathway; this is referred to as "*cross-presentation*". As a result, host immune systems generate immunity to exogenous agents and develop tolerance to self antigens. Therefore, cross-presentation is a typical source of indiscriminate presentation of self and foreign antigens [29]. The thymus is the control center where potentially aggressive T cells specific for autoantigens are eliminated and CD4^+^CD25^+ ^Treg cells that recognize autoantigens are selected. Observations on T cell antigen receptor (TCR) transgenic mice show that the thymus regulates the release of antigen-specific CD4^+^CD25^+ ^Treg cells into the peripheral circulation [30].

### Regulatory role of pro-inflammatory and regulatory cytokines in the onset of autoimmunity

As per the generally-accepted mechanism for the pathogenesis of autoimmune diseases, naive T cells upon activation by antigen produce IL-2 and then undergo clonal expansion and produce pro-inflammatory cytokines (tumor necrosis factor, TNF). CD4^+ ^T cells differentiate into at least two subsets of helper cells, T helper 1 (Th1) and T helper 1 (Th2). Th1 produce IFN-γ and lymphotoxin-α under regulation by IL-12, which activates the lymphocyte transcription factor STAT4 (signal transducer and activator of transcription 4) and plays a significant role in the onset of autoimmunity. In contrast, Th2 cells produce IL-4, which constrains cell-mediated immunity (CMI) and possibly inhibits the onset of autoimmune disease. Attenuation of the IL-10 and transforming growth factor-β (TGF-β) effect has been shown to result in inflammation and the onset of autoimmune diseases. In the context of lymphoid malignancy and autoimmunity, the absence of STAT3 from myeloid cells results in the onset of autoimmune diseases [31].

Therefore, the balance between proinflammatory (IL-2, IFN-γ and TNF) and regulatory (IL-4, IL-10 and TGF-β) cytokines probably determines any predisposition to develop autoimmune disease. IL-2 is known to mediate apoptosis through two different pathways, passive cell death (PCD) [32] and activation-induced cell death (AICD) [33]. PCD occurs mostly because crucial pro-survival signals are absent. Lack of cytokine signaling has been shown to cause an increase in mitochondrial permeability and cytochrome *c *release; along with apoptotic protease-activating factor 1 (APAF1), cytochrome *c *activates caspase-9 and downstream effector caspases. AICD, which is essential for the pathogenesis of autoimmunity and autoimmune lymphoproliferative syndrome, occurs mainly because of IL-2 mediated signaling via the death-domain-containing receptor Fas (CD95).

After the Fas-associated death domain (FADD) is activated, caspase-8 becomes active and downstream signaling results in cell death. Fas/Fas ligand (FasL; CD178) are deficient in autoimmune *lpr *and *gld *mice, which develop profound lymphadenopathies. The autoimmune lymphoproliferative disease (ALPS) found in humans is also consequent on mutations in Fas [33,34]. Furthermore, IL-2 has been shown to upregulate CTLA-4 (cytotoxic T-lymphocyte-associated protein 4; CD152), and CTLA-4-deficienciy leads to a fatal lymphoproliferative disease that is more aggressive than the lymphoproliferative disorders caused by either IL-2 or Fas deficiency [35]. However, abrogation or attenuation of chemokine induction (CXCL10, IP-10, inflammatory cytokines) at an auxiliary location would be likely to impair the onset of autoimmune disorder[36].

### Neo-antigens and molecular mimicry

The structures of host macromolecules and small molecules are markedly altered by acute or chronic oxidative stress and can behave as antigens ("neo-antigens"). Neo-antigens with sufficient homology or identity to host antigenic proteins prompt auto-reactivity. This phenomenon is referred to as "molecular mimicry". A detailed study demonstrating molecular mimicry linkages between viruses and host structures has been reported [37].

Drug metabolism is well known to generate neo-antigens in the form of protein adducts [38,39]. Potentially, chronic oxidative stress (COS) could be a slowly-evolving concomitant of the generation of mimetic neoantigens. Over time, COS could generate several adducted and/or non-adducted molecules that would essentially act as a "neo-antigens". This is consistent with the slow maturation of auto-antibodies in the evolution of autoimmune diseases. In practice, it is possible that more than one neo-antigens/autoantigens are involved in amplifying the autoaggressive lymphocytes by a process referred to as *"antigen spreading"*. This is an autoimmune reaction initially directed against a single autoantigen that spreads to other autoantigens, causing the T helper cells to recognize them [40].

### Molecular mimicry is most important cause of autoimmunity after viral infection

Molecular mimicry results in self-reactive T cells that are activated by cross-reactive ligands originating from infectious pathogens. It is of pivotal significance that molecular mimicry alone, in the context of infection, could not initiate an autoimmune disorder. However, in combination with various cellular factors (epitope avidity and extent of CD8^+ ^T cell activation), it may accelerate the process [41]. By utilizing distinct viral strains, it has been shown that T-cell receptor (TCR) affinity, and also negative regulatory molecules of host origin, are likely to play a crucial role in attenuating autoimmunity. It has further been suggested that autoimmune diseases *per se *are due to combinations of genetic and environmental factors. In particular, the affinity between the TCR and activating peptide-MHC ligand is essential; it might act as a limiting factor in eliciting an autoimmune response by molecular mimicry [42].

On the basis of these premises, it is argued that the amounts of genotoxic drugs and their adduct-forming metabolic derivatives probably play a pivotal role in accelerating autoimmune processes. Of course, they would act in combination with host-derived regulatory factors and the outcome would depend on the genetic predisposition of the individual. COS is highly likely to predispose an individual to an autoimmune disorder simply because the intrinsic defense mechanisms are depleted.

### "Bystander effect" and its role in the breakdown of self-tolerance: *a positive regulator of the onset of autoimmunity*

During COS, neo-antigens with target organ specificity potentially cause tissue damage and release a plethora of sequestered auto-antigens. This process is referred to as the "bystander effect". Such an outburst of autoantigens from the target tissue would potentially amplify the effect of the neo-antigens, leading to the breakdown of self-tolerance. To date, there is no definitive evidence that the host is either primed or programmed to bestow tolerance on the newly-evolving antigens resulting from COS.

### Chemical immunomodulation

Although the drugs used in clinical oncology are of low molecular weight, they cannot activate T cells but sensitize specific lymphocytes. Subsequent contact with a similar chemical or a metabolic derivative induces neo-antigens, which in combination with sufficient co-stimulatory signals cause the release of proinflammtory cytokines (e.g. IL-2, IL-7) and co-stimulatory mediators. This in turn causes the activation of neutrophils, monocytes, macrophages and complement pathways. It is reasonable to envisage that chemical activation of a leukocyte population releases pro-inflammatory cytokines, which in turn determine whether the host develops sensitivity or tolerance. It should be pointed out that drug-induced immune derangements are similar to those in graft-vs-host diseases [43].

### Immune response(s) to chemical stimulants

Owing to the overwhelming antigenic load in the host, naive T-cells (Th0) are activated and form Th1 or Th2 subpopulations. The Th1 response is characterized by the isotype specificity of the immunoglobulins formed (IgG2a and IgG2b), while the TH2 response is characterized by elevated levels of IgG1 and IgE. In most instances the initial APC is a dentritic cell (DC, CD80+, CD86+), but B-lymphocytes are the APCs for some chemicals that stimulate Th2 [44,45]. The immune modulation caused by a chemotherapeutic drug need not resemble the effects of its metabolic derivatives. It is therefore virtually impossible to determine the specific cause and effect relationship in a chemotherapeutic drug-induced autoimmune disease [46].

### Chemically-induced co-stimulatory signals

The following evidence supports the notion that COS plays a role in autoimmunity. Protein adducts generated as a result of oxidative metabolism of 2-bromo-2-chloro-1,1,1,-trifluoroethane (TFA-protein adducts) in CYP450 2E1 induce fulminant autoimmune-mediated halothane hepatitis [47]. TFA-protein adducts are structurally similar to the pyruvate dehydrogenase complex (PDC), which was identified as an autoantigen causing primary biliary cirrhosis, resulting in progressive destruction of the bile ducts [48]. Since sub-clinical primary biliary cirrhosis (PBC) is not easily diagnosed, the pathogenesis of PBC subsequent to the immune response to TFA-protein adducts or PDC might be due to acceleration of a pre-existing sub-clinical PBC. Protein modifications that lead to the formation of ("non-self") neo-antigens induce halothane hepatitis. At present there is no direct evidence to implicate TFA-adducts in co-stimulation *per se*.

### Role of metabolites/metabolic intermediates in the onset of autoimmunity

Aberrant induction of self-tolerance, chromatin-reactive T cells, auto-antibodies to chromatin, and inhibition of unresponsiveness to low-affinity auto-antigens (self-components) in the thymus during positive selection are characteristic effects of procainamide (procainamide-hydroxylamine) metabolites [49]. Administration of anti-CTLA-4 (blocking Ab) in mice that are susceptible to mercuric chloride (HgCl_2_) induced autoimmunity causes an increase in anti-nucleolar auto-antibodies. In DBA/2 mice, which are resistant to heavy metal-induced autoimmunity, similar treatment leads to the production of anti-nucleolar Abs, thus overcoming the genetic configuration of autoimmunity [50].

### Antigen spreading and its role in autoimmunity

Antigen spreading is significant in autoimmunity induced in a mouse model by xenobiotics (procainamide, mercuric chloride and gold (I)). Adoptive transfer of CD4^+^CD25^+ ^T cells from the xenobiotic-treated mice to untreated mice inhibits the formation of antinuclear autoantibodies. On the basis of these observations it was suggested that the T cell reactivity induced by the xenobiotic treatment may spread from xenobiotic-induced, nucleoprotein-related neoantigens to peptides of unaltered nucleoproteins [51].

### Toll-like receptors and autoimmunity

The toll-like receptors (TLRs) are a germ-line-coded receptor family that plays a pivotal role in innate immunity in a wide spectrum of organisms from insects to mammals. The innate immune response mechanism is either initiated or activated by structures referred to as pathogen-associated molecular patterns (PAMP), which are recognized by corresponding pattern recognition receptors (PRR). The best-characterized PAMPs are lipopolysaccharides (LPS), peptidoglycans, mannans, bacterial DNA and double-stranded bacterial RNA. Macrophages, B-cells and dendritic cells (DC) express PRR. PRR are classified into three specific types: secreted, endocytic and signaling. Mannan binding lectin represents the secreted type, while the macrophage mannose receptor belongs to the endocytic class and the toll-like receptors (TLRs) are signaling types [52].

TLRs are essential for detecting PAMPs, and this has been identified as the first line of defense for pathogen recognition, for which a range of antimicrobial products and numerous proinflammatory cytokines are generated by the host. The *Drosophila *protein Toll that is required for mounting an effective immune response to *Aspergillus fumigatus *has been identified as a lipopolysaccharide (LPS) receptor. It plays a pivotal role in the primary recognition of infectious pathogens by mammals [53].

TLRs are divided into five subfamilies on the basis of amino acid sequence homology: TLR-1, 2, 6, 10; TLR-3; TLR-4; TLR-5; and TLR-7, 8, 9. Structurally, the extracellular region of a TLR contains leucine-rich repeats flanked by cysteine-rich motifs; a TOLL/IL-1 receptor (TIR) homology domain in the cytoplasmic region is critical for signaling. Given the sequence similarities between the TIR domain and the cytoplasmic tails of IL-1 and IL-18 receptors, it has been suggested that their signaling sequences are similar (see box 12-1 and Figure 12-3 of [54].

Antigen (epitope-specific) recognition by B-cell receptors (BCR) induces signals that cause B cell proliferation and antibody production. Concurrent recognition by CD4 (T-helper) cells generates specific cytokines that are essential for antigen-specific antibody production by B-cells. It has been conclusively demonstrated that loss of tolerance to a given antigen by both B and T-cells is a primary cause of autoimmune reactions [55]. B-cells are known to generate anti-self IgG2a antibodies of low affinity. However, these IgG2a can be recognized as immunogenic by B-cells and as PAMP by TLRs, thus inducing autoantibodies to nuclear antigens [56].

Concurrent activation of BCR and B-cell TLR-9 by such IgG2a is due to recognition as non-self and results in the formation of a self DNA (autoantigen)-IgG2a (autoantibody) immune complex. Binding of such a complex to BCR triggers endocytosis, causing effective delivery of the denatured chromatin fragments to endosome-associated TLR-9. Activation of TLR-9 by exogenous or endogenous CpG-DNA in MLR-Fas *lpr/lpr *mice induces the progression of renal diseases [57]. It should be pointed out that MyD88-dependent receptor activation is required for the formation of autoantibody-autoantigen immune complexes in adaptive immune responses [58].

Dendritic cells (DCs) engorged with a cardiac muscle-specific self (autoantigen) peptide caused CD4^+^-cell-mediated autoimmune myocarditis, which progresses to dilated cardiomyopathy and heart failure. It has been suggested that this is a TLR-dependent process. Formation of the self-peptide-loaded DCs may have been provoked by various microbial epitopes acting via TLRs during chronic infection [59]. One such factor may be uric acid. Studies on DC maturation have shown that uric acid is a major endogenous danger signal from injured cells. It induces DC maturation in the presence of antigen and significantly up-regulates the generation of responses originating from CD8^+ ^T cells [60].

Thus, in the context of the hypothesis proposed here (see below), it is possible that a host overloaded with one or more antigenic determinants (epitopes) from one or more infectious agents causes stress that in turn activates TLRs. It is contended that by a yet uncharacterized mechanism, this could lead to multi-drug resistance during the course of treatment for these infections, and this could develop into autoimmunity.

However, the free radical theory of autoimmunity proposed here differs markedly from the "danger theory" of immune activation proposed by Matzinger. Essentially, this states that the host immune system does not differentiate between self- and non-self- but mounts an effective immune response only to danger signals originating from necrotic or stressed cells [61].

**Figure 1:**
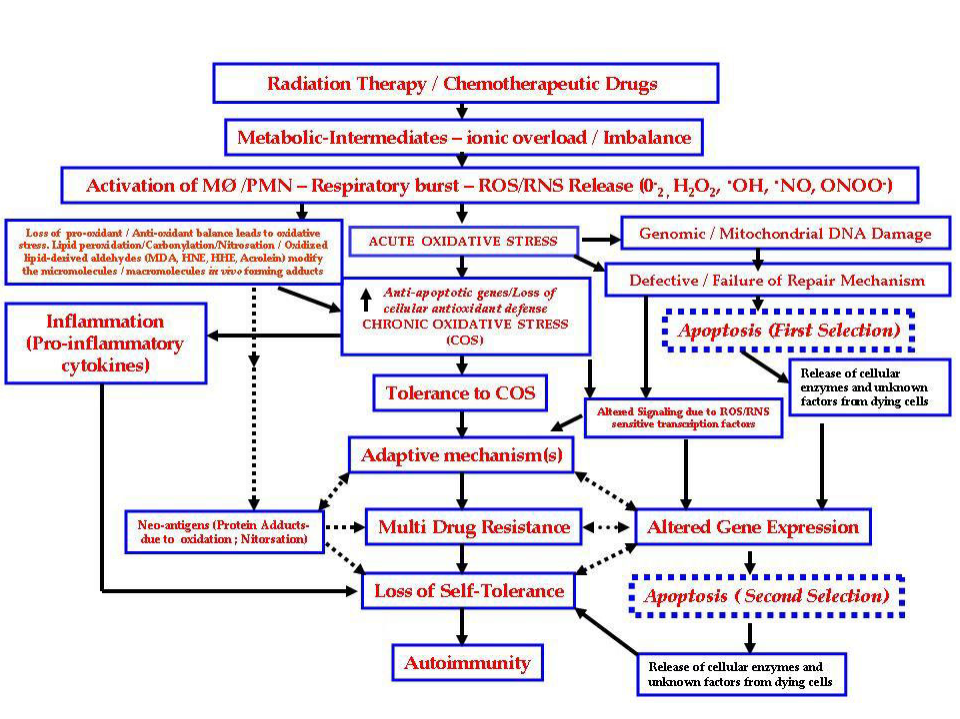


In support of my contention, it has been proposed that macrophages and DCs express TLR on their surfaces so they can recognize PAMP and initiate appropriate signals for inducing reactive oxygen and nitrogen intermediates. Because this would also activate APCs by inducing pro-inflammatory cytokines and up-regulate co-stimulatory molecules for the activation of TLRs (see Figure 1, 2 and Table 1 of [62]), it has been proposed that one possible mechanism for the onset of autoimmunity is mediation of the breakdown of peripheral tolerance by hyperactive APCs, causing the activation of autoreactive cells [63].

I contend that autoimmunity resulting from COS is not an all-or-none response but an evolutionary process that engulfs the host immune system over a period of time. Arguably, a danger signal could play a pivotal role in the onset of autoimmune disorders, but this alone could never account for the collapse of the host immune response. For an effect of such magnitude, the host must endure diverse stress signals that eventually lead to the collapse of tolerance and trigger autoimmune reactions.

Although a particular TLR is responsible for ligand-induced signaling, the TLR repertoire that confers ligand-binding and signaling specificities results from heterodimerization and the participation of diverse non-TLR adaptor molecules. Several adaptor molecules have been identified and prominent among these are myeloid differentiation factor 88 (MyD88), MyD88-adaptor-like/TIR-associated protein (MAL/TIRAP), Toll-receptor-associated activator of interferon (TRIF) and Toll-receptor-associated molecule (TRAM). They transduce signals from all regions homologous to the Toll/interleukin-1 receptor (IL-1R) (TIR) domain. Such signals activate intracellular protein kinases, which in turn activate transcription factors that up-regulate inflammatory cytokine and fibrotic genes (see Figure 1 in [64]). The function of a fifth adaptor, SARM (sterile alpha and HEAT/Armadillo motif protein), has yet to be defined [64]. The molecular basis of the TLR-associated signaling cascade has been discussed in detail elsewhere [65].

TLR4/MD-2 and RP105/MD-1 signal the presence of LPS in the host. RP105 is expressed on virtually all mature B-lymphocytes. RP105/MD-1-mediated signaling induces B-cell proliferation and the pathogenesis of systemic lupus erythematosus (SLE) [66]. It has been suggested that TLR9 activation triggers systemic autoimmunity and contributes through adaptive and innate immune mechanisms to the CpG-DNA-induced succession of lupus nephritis [67].

Activation of naïve polyclonal B cell proliferation by TLR7 ligands such as *resiquimod (R848) *and *loxoribine *requires the presence of plasmacytoid dendritic cells (PDCs), whereas similar activation via the TLR9 ligand CpG is independent of PDCs. Also, in the presence of type I interferon (IFN), ligation of TLR7 triggers the multiplication of polyclonal B cells and their subsequent differentiation toward Ig-producing plasma cells. This process occurs independently of T and B cell Ag [68].

TLR-9 induced lupus B cell activation has been shown to modulate T-cell mediated inflammatory reactions through IL-10. Furthermore, B-cell mediated lupus pathogenesis can be mediated by B cells acting as APCs for auto-antigens and autoantibody-producing effector cells. B-cells are also sources of IL-10 [69].

Cross-presentation of peripheral self-Ags by DC can induce deletion of auto-reactive CTL by cross-tolerance. Activation of tolerogenic DC may cause autoimmunity by stimulating autoreactive CTL. It was concluded that DC activation by TLR ligands is insufficient to break peripheral cross-tolerance in the absence of specific CD4^+ ^T helper cells, so autoimmunity is promoted by stimulating the early effector phase of autoreactive CTL only when their precursor frequency is extremely high [70]

### Cause and effect of COS in the onset of autoimmunity

Substantial improvement in the therapeutic regimen increases the survival rate for cancer patients (62% of adult and 77% of pediatric cancer patients survive beyond 5 years). It is therefore theoretically possible that the lingering effects of radiation therapy and chemotherapeutic drugs, or a combination of both, impart slow but finite damage to non-cancerous tissues or cells. This condition could therefore be described as a chronic disease, and the late effects of radiation and chemotherapy on normal tissues/cells remain a significant health risk [71].

Upon exposure to genotoxic agents, bioactive metabolites are formed *in vivo*. These metabolites as well as the parent compound damage subcellular components in a target organ-specific manner. The extent of such damage depends on the concentrations of metabolic intermediates or parent compounds and/or both. Exposure to toxic chemicals can cause increased iron accumulation in the spleen as a result of erythrocyte damage and free iron release in the target organ. Accumulation of free iron has been shown to cause the activation of phagocytic cells (macrophages, neutrophils) and subsequent release of reactive oxygen and nitrogen species *in vivo *[72-74].

The late effects of radiation and chemotherapeutic drugs (and genotoxic compounds) on normal cells would lead to activation of stress-response kinases (mitogen-activated stress kinases – MAPK) and redox-sensitive transcription factors, and up-regulate pro-inflammatory cytokines [75,76]. The continued and ever-increasing presence of chemotherapeutic drug metabolites, in combination with substances released from the damaged tissues, are potential sources of as yet uncharacterized toxins. It is possible that these compounds could cause an aberrant (chronic inflammatory) response *in vivo *[77].

In such pathophysiological states, peripheral blood leukocytes would probably be activated, leading to a sequential respiratory burst releasing ROS (H^•^, ^•^OH, O_2 _^•- ^and H_2_O_2_). Owing to its inherent instability and reactivity, ^•^OH reacts with biological molecules in its vicinity within 10^-9 ^s of its formation [78]. In addition to ROS in pathophysiological states, iNOS, the inducible or calcium-independent isoform of nitric oxide synthase, mediates the formation of di-nitrogen trioxide (N_2_O_3_) and peroxynitrite (O = NOO-) (RNOS), causing nitrosative and oxidative stress *in vivo *[79] and altering signaling cascades with effects on the regulation of gene expression [80,81].

Protein nitration occurs as a result of oxidative stress. Tyrosine residues are nitrated via the peroxynitrite-mediated pathway, and haem-containing peroxidase catalyzed reactions also occur. Nitrite (NO_2 _^-^) is an end-product of NO metabolism that can be oxidized by haem peroxidases (e.g. horseradish peroxidase, lactoperoxidase and myeloperoxidase) forming the reactive nitrogen species NO_2_. Nitrite also reacts with tyrosine residues causing the nitration of proteins. NO_2 _reacts with HOCl via a myeloperoxidase-catalyzed reaction between H_2_O_2 _and Cl^-^, forming nitryl chloride (NO_2_Cl). NO_2_Cl also nitrates protein tyrosine residues.

S-Nitrosylated proteins are formed when cysteine thiol groups react with nitric oxide (NO) in the presence of an electron acceptor to form an S-NO bond. Nitrated proteins are protected from reductive or transnitrosative degradation by storage in membranous structures (e.g. lipophilic protein folds, vesicles and interstitial spaces). Caspases are typically sequestered in an S-nitrosylated (inactive) form within the intermembrane spaces of mitochondria. Appropriate apoptotic stimuli (e.g. Fas-Fas ligand binding) release caspases into cytosol where they are denitrosylated to initiate apoptosis. Hypoxia-inducible factor I (HIF-1), stimulating proteins 1 and 3 (Sp1 and Sp3), nuclear factor-κB (NF-κ B) and the prokaryotic transcription factor OxyR are also affected by S- nitrosylation.

ROS/RNOS are transient, necessitating the use of reaction products as biomarkers or indices of nitrosative and oxidative stress *in vivo*. Oxidized products such as thiobarbituric acid reaction products (TBARs), 4-hydroxynonenal (4-HNE) and hexane are routinely considered markers of lipid peroxidation in both animal models and patients [82]. Furthermore, sustained nitrosative stress (increased nitrotyrosine formation) has been observed post-irradiation [83,84]. Increased lipid peroxidation has also been reported in patients developing radiation pneumonitis [85]. Hypoxia was identified in the rat lung 6 weeks after a single dose of 28 Gy using the hypoxia marker pimonidazole [86]. During hypoxia, increased ROS/RNOS production and reduced antioxidant and antioxidant enzyme production have been observed [87-89].

Sustained ROS/RNOS accumulation leads to COS *in vivo*. Although the precise mechanisms involved are not known at present, possibilities include reduced levels of the antioxidant vitamins C and E [90,91], differential regulation of the xanthine oxidoreductase system [92] and/or aberrant arachidonic acid metabolism [93].

Changes in protein conformation can cause aggregation and accumulation in tissues *in vivo*. Metabolic activities (possibly oxidative stress/nitrosative stress) can lead to sustained changes in the levels of metal ions, chaperone proteins and pH, leading to macromolecular crowding and increasing the concentration of misfolded proteins in the intracellular milieu. These changes have a significant role in protein aggregation and consequent loss of functional properties. It is equally possible that proteins with altered conformation have toxic effects in the intracellular milieu [94].

Despite the lack of direct evidence, it is feasible that proteins with altered conformations may present sequences similar to host self-antigenic determinants (epitopes) and therefore play a role in neo-antigen formation, thus perturbing the host immune system and potentially contributing to the evolution of multidrug resistance-induced autoimmunity.

As suggested earlier, pro-inflammatory cytokines (IL-2) probably contribute to the loss of self-tolerance and possibly to the formation of neo-antigens. It is conceivable that in malignant disorders, cells that are subjected to stress (oxidative/nitrosative) become unresponsive to ever-increasing doses of ionizing radiation or chemotherapeutic drugs because of their altered state. Thus, the patient would lose pre-existing antioxidant defense mechanisms, causing limited or no antioxidant defense. This would pave the way for the loss of drug sensitivity and multi-drug resistance, and also the generation of COS.

In summary, the aforementioned circumstances create a state of imbalance between ROS/RNOS generation and removal *in vivo*, thereby causing oxidative/nitrosative stress. My contention is that the intracellular environment, as outlined above, would be suitable for deregulating the immune mechanism(s) in the cancer patient. Autoimmune mechanisms(s) would subsequently be triggered, causing loss of self-tolerance and the development of autoimmune disorders.

### Activation of antioxidant mechanisms as cause of drug resistance: a critical appraisal

In essence, the multi-drug resistance (MDR) phenotype of a given clinical tumor burden results from a combination of factors including decreased uptake of cytotoxic anti-neoplastic drugs and alterations in intracellular metabolism impairing the capacity of such drugs to kill cells. As a result, tumor cells do not adequately control the cell cycle, and there is increased repair of DNA damage, decline in apoptotic cell death and deregulated energy-dependent efflux of cytotoxic drugs across the plasma membrane [95].

Overexpression of Pgp, MDR-associated-protein 1 (MRP1/ABCC1) or ABCG2 has been positively correlated with the evolution of MDR as well as cross-resistance to structurally unrelated anti-neoplastic drugs in a clinical tumor burden [96]. Of the several mechanisms suggested to underlie the onset of MDR, ABCC1 (MRP1) and its homologues ABCC2 (MRP2), ABCC3 (MRP3), ABCC6 (MRP6) and ABCC10 (MRP7) mediate the transport of glutathione (GSH), glucuronate or sulfate conjugates of organic anions [97]. Inhibitors of Pgp have been evaluated as effective therapeutic measures for blocking the efflux of chemotherapeutics used in clinical oncology.

Owing to the heterogeneity of a tumor burden (in particular colon, kidney or adrenocortex) endowed with both Pgp- and non-Pgp-dependent mechanisms that cause MDR, inhibitors of Pgp are of limited scope as adjuncts to chemotherapy. Furthermore, the unexpectedly high mortality rate has been attributed to intolerable tissue toxicity that is partly due to the elevated plasma concentrations of Pgp inhibitors [98]. An ideal chemotherapeutic transporter antagonist with high transporter affinity and low pharmacokinetic interaction with non-related drugs, which would restore the efficiency of treatment in MDR tumor burdens with no or minimum cytotoxicity, has yet to be formulated [99]. On the basis of these observation it is suggested that in a given clinical tumor burden, "intrinsic MDR" and "acquired MDR" might coexist, making existing therapeutic interventions unable to prevent relapse.

### Oxidative stress and MDR

Chemosensitizers as an adjunctive components in combination therapy (chemotherapy) have a variable therapeutic index. This approach is also known to generate oxidative stress owing to the accumulation of ROS and RNS in the intracellular milieu of a given MDR tumor. It has been suggested that oxidative stress-induced apoptosis is a plausible cytotoxic effect of chemotherapeutics in a MDR tumor burden [100-104].

### Anti-oxidative (enzymatic and non-enzymatic) system and MDR

Antioxidants confer protection against oxidative stress by quenching free radicals, chelating redox metals and interacting with (and regenerating) other antioxidants within the "antioxidant network". When optimal concentrations are sustained in tissues and biofluids they can function in both the aqueous and membrane domains. The most efficient enzymatic antioxidants are superoxide dismutase, catalase and glutathione peroxidase [105]. Non-enzymatic antioxidants include vitamins C and E, carotenoids, thiols (glutathione, thioredoxin and lipoic acid), natural flavonoids and melatonin [106]. Few antioxidants can regenerate other antioxidants to restore the reduced intracellular state via an "antioxidant network". The redox cycles of vitamins E and C have this capacity, driven by the redox potentials of the [Red/Ox] couple [107]. Antioxidants attenuate ROS by binding to transition metal-containing proteins, transferrin or ceruloplasmin, inhibiting cellular reactions (Vitamin E) and detoxifying ROS and RNS, (GSH, SODs, catalase) [108].

### Role of thioredoxin (Trx)/thioredoxin reductase (TrxR) system in multi-drug resistance

Overexpression of thioredoxin reductase (TrxR) increases growth rates and resistance to cytotoxic agents that induce oxidative stress [109,110]. Detoxification of ROS and up-regulation of antioxidant genes are plausible mechanisms [111]. In tumor cells, the thioredoxin (Trx)/thioredoxin reductase (TrxR) couple produces a reduced form of extracellular Trx, which acts as a growth factor conferring protection from the NK-lysin, tumor necrosis factor-α and the T-lymphocyte respiratory burst [112]. Effective inhibition of the Trx/TrxR system increases the sensitivity of tumor cells to chemotherapeutic compounds [113-115]. Thioredoxin and peroxiredoxin 1 are up-regulated in drug-resistant breast cancer patients who are clinical non-responders to docetaxel [116].

### Role of the glutaredoxin reductase (GR)/glutaredoxin (GRX) system in drug resistance

Increased activity of antioxidants such as catalase, glutathione peroxidase and DT-diaphorase, and increase in glutathione level, are associated with the onset of resistance in Chinese hamster cells chronically exposed to menadione [117]. Increased expression the α,μ and π isoenzymes of GST might confer a multidrug-resistant phenotype on rat hepatic preneoplastic nodules [118]. Increases in gamma-glutamyl transpeptidase (GGT) and gamma-glutamylcysteine synthetase (GCS) have a role in the acquired resistance to quinone toxicity in rat lung epithelial cells [119]. Elevated GST activity might also contribute to the conversion of breast tumors to a tamoxifen-resistant phenotype [120].

Development of resistance to doxorubicin in human erythroleukemia cells is correlated with an increase in GSH and GST and related enzymes (glutathione peroxidase, glutathione reductase) [121]. Genomic amplification resulting in an increase in GST-π expression has been observed in head and neck squamous cell carcinoma cell lines models and also in clinical tumor burdens resistant to cisplatin. Clinical reports confirm mortality in patients with GST-π amplification in the entire tumor burden subsequent to chemotherapy [122]. GST-π been shown to attenuate the formation of the 7-(2-oxo-hepyl)-substituted 1, N(2)-etheno-2'-deoxyguanosine adduct with 2'-deoxyguanosine in human colonic cancer cells that are resistant to anticancer drugs [123].

siRNA-mediated down-regulation of GRX-2 in HeLa cells induces an increased sensitivity to doxorubicin and phenylarsine oxide. In normal HeLa cells, exposure to non-lethal oxidative stress causes an increase in endogenous GRX-2. This has been implicated in protection against toxic compounds that induce oxidative stress [124].

CAL1 human melanoma cells overexpress GST-μ1 after exposure to anticancer drugs (vincristine, chlorambucil). A concurrent increase in both GST-μ1 and MRP-1 might have a role in protection against vicristine-mediated cytotoxicity [125]. Also, it has been suggested that GRX-2 is pivotal in the glutathionylation and deglutathionylation of proteins via multiple signaling pathways in a wide range of GSH/GSSG ratios associated with different cellular redox states [126]. The transcription factor Nrf2 mediates the up-regulation of γ-GCS and GSH synthesis, and induces resistance to Imatinib (BCR/ABL tyrosine kinase inhibitor) in chronic myelogenous leukemia [127].

Activation of glutathione peroxidase and glutathione reductase was observed in the onset of radio resistance and cross-resistance to chemotherapeutic agents in glioblastoma [128]. Increased binding of AP-1 (activator protein-1) activity, observed in an electrophoretic mobility shift assay, was suggested as the molecular mechanism by which GST is overexpressed, in turn conferring resistance to doxorubicin in leukemia [129].

Overexpression of GRX-2 in HeLa cells confers a significant antiapoptotic and pro-survival effect upon exposure to doxorubicin and phenylarsine oxide, possibly via inhibition of cytochrome c release [130]. Pancreatic cancer cells exposed to triterpenoid 2-cyano-3,12-dioxooleyl-1,9-diene-28-imidazolide (CDDO-Im) caused the depletion of mitochondrial glutathione, leading to apoptosis [131].

A suggested downstream target of redox-sensitive signaling is ribonucleotide reductase, which is likely to play a significant role in the antioxidant-mediated protection of tumor cells [132]. Glutathione peroxidase 1 and GST-π1 are up-regulated in breast cancer patients who are clinical non-responders to docetaxel but show drug resistance [133].

### Role of redox-sensitive signaling proteins and MDR

#### Metallothioneins (MTs)

MTs are zinc-binding protein thiols with antioxidant attributes that increase in tumor cells. This observation suggests that overexpression of MTs is significant in the acquisition of drug resistance in human tumor cells [134].

#### Superoxide dismutase (SOD)

A significant increase in SOD may be involved in the conversion of breast tumors to a tamoxifen-resistant phenotype [120]. Mn-SOD-dependent activation of the zinc-dependent matrix metalloproteinase family (MMP-1,-2) has been positively correlated with an increased incidence of metastasis in gastrointestinal tumors [135]. Also, increased Mn-SOD activity confers resistance to doxorubicin in human erythroleukemia cells [121]. It has been suggested that up-regulation of SOD is significant in the onset of radioresistance and cross-resistance to chemotherapeutic agents in glioblastoma [136].

#### Vitamin C

The role of Vitamin C in decreasing the incidence of stomach, lung and colorectal cancer may be attributable to the inhibition of *N*-nitroso compound generation [137].

#### Flavonoids

A regular intake of flavonoids such as polyphenols and quercetin is linked to lower incidences of gastrointestinal and also lung and breast cancer [138].

#### Selenium

Se (200μg/day) seems to reduce the incidences of lung, colon and prostate cancer [139,140]. Increased levels of antioxidant enzymes (SOD, catalase, glutathione peroxidase) and non-enzymatic antioxidants (GSH, vitamin C, thioredoxin) are significant in several clinical tumor burdens [141].

Collectively, antioxidants confer a reducing intracellular environment enabling tumor burdens with MDR to evade apoptosis and acquire growth advantage with increased cell survival signals [142]. For these reasons, the "redox buffering" capacity of a given tumor burden is a potential therapeutic target for effective cancer-preventive and therapeutic drug design [143].

#### Summary

Oxidative stress induces conformational changes in intracellular proteins containing cysteine residues, triggering ionization of the sulfhydryl moiety (-CH_2_SH to -CH_2_S^-^). Pro-survival genes are up-regulated in a reduced intracellular state. It is suggested that increased expression of pro-survival genes enables tumor cells to evade chemotherapeutically-induced cytotoxicity, conferring an adaptive growth advantage that aggravates the tendency of tumor cells with MDR to repopulate the tumor burden. This leads to relapse and the subsequent development of autoimmunity via several chronic redox state-dependent reaction cascades.

### Hypothesis

The following proposal (illustrated in Figure 1) accounts for the role of COS as an essential element in the evolution of drug resistance-mediated induction of autoimmunity. The hypothetical scheme is based on the premises outlined above, and defines a possible sequence of events beginning from radiation therapy/chemotherapy and leading to the evolution of autoimmune disorders.

As shown in Figure 1, the toxicity consequent on radiation therapy/chemotherapy is due to the metabolic derivatives and/or ionic overload (imbalance). These, in turn, induce the activation of phagocytes and the associated respiratory burst causing the release of ROS and RNS. Lymphocytes (T and B cells) that are subjected to oxidative stress are more sensitive to such stress. Lymphocytes exposed to H_2_O_2 _exhibit extensive genomic damage (DNA strand breaks). Oxidative/nitrosative stress-induced alteration in the host lymphocyte genome is likely to engender aberrant cellular pathways that could lead to rapid cell lysis (necrosis) or programmed cell death (apoptosis). Owing to the overwhelming ROS/RNS, the antioxidant reserve may be depleted or inactivated by one or more mechanism(s). Such an intracellular environment would be conducive to lipid peroxidation and accumulation of oxidized lipid-derived aldehydes.

Lipid peroxidation-derived aldehydes are a potential source of protein modification (oxidation resulting in carbonylation and nitrosation). This phase could be classed as a state of acute oxidative stress. It is recognized that acute oxidative stress causes genomic and/or mitochondrial DNA damage. As a result, it is conceivable that pro-apoptotic gene expression would be up-regulated. Should this become overwhelming, the tumor cells are programmed to die by apoptosis **(First Selection)**. These processes would be exacerbated if the DNA repair mechanisms *in vivo *failed or were defective.

It is possible that during acute oxidative stress, anti-apoptotic proteins/drug resistance genes may be overexpressed in a select but limited number of tumor cells. It is also possible that those cells may have endured minimal or no loss of anti-oxidant defense. The select cell population would therefore survive and eventually cope with the ensuing COS. Such a pathophysiological state would obviously lead to inflammation, engendered by inflammatory cytokines and tumorigenic chemokines, which in turn prompt deregulation of the immune defense mechanisms.

Should there be a loss of antioxidant defense or failure of adaptive mechanisms, or both, vulnerable tumor cells would be programmed to undergo apoptosis **(Second Selection)**. Essentially, COS acts as a regulator for selecting the most tolerant tumor cell population, which is adapted to survive radiation/genotoxic insults. But there are no adequate signaling mechanism(s) in these aberrant cells, impairing their ability to sustain homeostasis.

The COS-induced impairment of the intracellular metabolic machinery again causes sustained genomic DNA damage and potentially depletes the antioxidant reserve. This may occur either concurrently or sequentially and lead to the multi-drug resistant genotype, as well as facilitating the formation of neo-antigens. It has previously been hypothesized that, owing to the loss of tolerance to autoantigens and sustained accumulation of neo-antigen(s), an autoimmune response would be likely to evolve [144,145].

A factor that has frequently been overlooked in the *in vivo *context is that cells undergoing apoptosis or necrosis release considerable amounts of proteolytic enzymes and other mediators. It is conceivable that these factors would be detremental to normal as well as multi-drug-resistant tumor cells. They would cause havoc in intracellular signaling, contributing to the alteration in gene expression. It is also possible that these factors would lead to cell death via either apoptosis or necrosis **(Second Selection)**.

Those normal cells that acquire tumor-resistant genotypes are successful in passing through all the selection processes and would eventually emerge as multidrug-resistant cells. Under these circumstances, accumulation of neo-antigens or other inflammatory cytokines would also decimate the host immune defenses. These events lead to an intracellular milieu conducive to a lethal breakdown in host self-tolerance. This, in turn, causes a surge in immune disorders, leading to the sequence of events that result in autoimmunity.

Should this effect prevail, it would be a potential cause of death in a cancer patient. Therefore, an effective means of treating the lymphoproliferative disorders that result from failure of therapeutic measures is to delineate the precise molecular mechanisms leading to COS and the consequent genomic DNA damage and expression and regulation of repair proteins. Only a combination of therapeutic measures can attenuate or disengage the pathophysiological consequences of these deleterious COS-induced effects on tumor cells.

In summary, these COS-induced effects would directly (i) alter signal transduction and (ii) induce epigenetic mechanisms (hypo/hyper-methylation; hypo/hyper-acetylation of the elements that regulate anti-apoptotic or pro-apoptotic genes) [146-151]. In the context of COS as a regulatory element in the evolution of autoimmunity, alterations in cytokines (IL-2 expression) would exert the most significant impact next to the neoantigens. Therapeutic measures directed at modulating such epigenetic mechanism(s) in a development stage-specific manner (lymphoproliferative disorder) would potentially attenuate the impact of drugs and the subsequent evolution of autoimmune disorders.

## Competing interests

The author(s) declare that they have no competing interests.

## Note

*In honor of my mother Srimathi Kannika Kannan

Chronic oxidative stress is an essential regulatory element in the evolution of drug resistance-mediated induction of autoimmunity
